# Complete Genome Sequence of *Pseudomonas aeruginosa* Phage-Resistant Variant PA1RG

**DOI:** 10.1128/genomeA.01761-15

**Published:** 2016-02-18

**Authors:** Gang Li, Shuguang Lu, Mengyu Shen, Shuai Le, Yinling Tan, Ming Li, Xia Zhao, Jing Wang, Wei Shen, Keke Guo, Yuhui Yang, Hongbin Zhu, Shu Li, Junmin Zhu, Xiancai Rao, Fuquan Hu

**Affiliations:** Department of Microbiology, College of Basic Medical Science, Third Military Medical University, Chongqing, China

## Abstract

Bacteria have evolved several defense systems against phage predation. Here, we report the 6,500,439-bp complete genome sequence of the *Pseudomonas aeruginosa* phage-resistant variant PA1RG. Single-molecule real-time (SMRT) sequencing and *de novo* assembly revealed a single contig with 320-fold sequence coverage.

## GENOME ANNOUNCEMENT

*Pseudomonas aeruginosa* is a Gram-negative bacterium distributed in highly diverse ecological niches, such as soil, water, and various living host organisms (1, 2). As an opportunistic pathogen, *P. aeruginosa* causes significant morbidity and mortality among compromised individuals and is associated with hospital-acquired pneumonia, urinary tract infections, surgical site infections, and chronic cystic fibrosis (CF) lung infections (2, 3). Due to notable biofilm formation and intrinsic drug resistance, it is very difficult to treat *P. aeruginosa* infections with antibiotics in the clinical setting (4, 5).

Bacteriophages (phages) are the most abundant and most diversified microorganisms on the planet (6). The arms race between phages and bacteria has been an important factor driving bacterial evolution and diversification (7). Bacteria have evolved several defense systems against phage predation, such as adsorption inhibition, restriction-modification systems, abortive infection systems, and clustered regularly interspaced short palindromic repeat (CRISPR)-CRISPR-associated (Cas) systems (7). Bacterial phage-resistant variants can easily be obtained under lab conditions (8), and besides being resistant to phage infection, they usually show changes in phenotype, including biofilm formation, virulence, and small-colony variants (SCVs) (9–11). The isolation and characterization of phage-resistant variants might provide extensive understanding not only of bacterial defense systems but also of the complicated interactions between bacteria and phages.

The host bacterium of lytic phage PaP1 (12) is *P. aeruginosa* PA1, a clinical isolate (13). A phage-resistant variant of *P. aeruginosa* PA1 was obtained from phage PaP1 lysates and named *P. aeruginosa* PA1RG. The genomic DNA of *P. aeruginosa* PA1RG was extracted from the overnight cultures, grown in LB broth, and purified using the Wizard genomic DNA purification kit (Promega, WI). PacBio single-molecule real-time (SMRT) sequencing of the PA1RG genome was carried out at the Institute of Medicinal Plant Development (IMPLAD) (Beijing, China) using the PacBio RSII instrument (Pacific Biosciences, Menlo Park, CA, USA) (14, 15). Libraries of 5 kb were constructed, and 4 SMRT cells of the libraries were sequenced with 90-min movies. *De novo* assembly was performed using RS_HGAP_Assembly version 2.0 (16), revealing a single contig with 320-fold sequence coverage. The length of the PA1RG genome is 6,500,439 bp, with an average G+C content of 66.34%. Genome annotation of *P. aeruginosa* PA1RG was performed using the NCBI Prokaryotic Genome Annotation Pipeline (17) (2013 release; http://www.ncbi.nlm.nih.gov/genome/annotation_prok/).

### Nucleotide sequence accession number.

The complete genome sequence of *P. aeruginosa* strain PA1RG has been deposited in GenBank under the accession no. CP012679. The version described in this paper is the first version.
